# Regulation of expression of two LY-6 family genes by intron retention and transcription induced chimerism

**DOI:** 10.1186/1471-2199-9-81

**Published:** 2008-09-25

**Authors:** Vincenzo Calvanese, Meera Mallya, R Duncan Campbell, Begoña Aguado

**Affiliations:** 1 Centro de Biología Molecular Severo Ochoa (CBMSO), CSIC, Madrid, 28804, Spain; 2 MRC Rosalind Franklin Centre for Genomics Research, Cambridge, CB10 1SB, UK; 3 Department of Physiology, Anatomy and Genetics, University of Oxford, Oxford, OX1 3QX, UK; 4 Department of Medicine, University of Cambridge, Wolfson College, CB3 9BB, UK; 5 Centro Nacional de Investigaciones Oncológicas (CNIO), Madrid 28029, Spain

## Abstract

**Background:**

Regulation of the expression of particular genes can rely on mechanisms that are different from classical transcriptional and translational control. The *LY6G5B *and *LY6G6D *genes encode LY-6 domain proteins, whose expression seems to be regulated in an original fashion, consisting of an intron retention event which generates, through an early premature stop codon, a non-coding transcript, preventing expression in most cell lines and tissues.

**Results:**

The MHC LY-6 non-coding transcripts have shown to be stable and very abundant in the cell, and not subject to Nonsense Mediated Decay (NMD). This retention event appears not to be solely dependent on intron features, because in the case of *LY6G5B*, when the intron is inserted in the artificial context of a luciferase expression plasmid, it is fully spliced but strongly stabilises the resulting luciferase transcript. In addition, by quantitative PCR we found that the retained and spliced forms are differentially expressed in tissues indicating an active regulation of the non-coding transcript. EST database analysis revealed that these genes have an alternative expression pathway with the formation of Transcription Induced Chimeras (TIC). This data was confirmed by RT-PCR, revealing the presence of different transcripts that would encode the chimeric proteins CSNKβ-LY6G5B and G6F-LY6G6D, in which the LY-6 domain would join to a kinase domain and an Ig-like domain, respectively.

**Conclusion:**

In conclusion, the LY6G5B and LY6G6D intron-retained transcripts are not subjected to NMD and are more abundant than the properly spliced forms. In addition, these genes form chimeric transcripts with their neighbouring same orientation 5' genes. Of interest is the fact that the 5' genes (CSNKβ or G6F) undergo differential splicing only in the context of the chimera (CSNKβ-LY6G5B or G6F-LY6G6C) and not on their own.

## Background

In the post-genomic era biological endeavours are more and more centred on understanding the different mechanisms of regulation of gene expression. An increasing number of interacting regulatory levels are being explored and, in this amazing landscape, alternative splicing is even more interesting because, starting from a relatively limited number of genes, it is involved in increasing proteome complexity, [1-3]. In relation to this, alterations of splicing patterns or mis-splicing of genes are involved in several pathologies, [4-6] including several genetic diseases such as spinal muscular atrophy (SMA), myotonic dystrophy (MD), Alzheimer's disease (AD), and retinitis pigmentosa (for review see [7]). Aberrant splicing has also been linked to cancer ([8] and refs).

The human Major Histocompatibility Complex (MHC) is located at chromosome 6p21.3, and is ~4 Mb in length. It consists of three regions, the class I and class II regions flanking the central class III region. The class III region is ~0.9 Mb in length and contains 62–64 genes and 2–3 pseudogenes, depending on the haplotype [9,10]. Of the predicted genes, at least 24 (41%) have a definite or potential role in the immune system. The human MHC has been linked to susceptibility to many diseases, and often these associations cannot be fully explained by variation in the class I and II genes [10,11]. Therefore, the study of the class III region genes, especially the novel genes with a potential role in the immune system, may provide insights into the understanding of these diseases. Transcriptome studies of some MHC class III region genes indicate a high rate of different splicing events. Previously, we have defined precisely the alternative splicing patterns of a cluster of five genes of the Lymphocyte antigen-6 (LY-6) superfamily [12] and characterised the expression of the corresponding proteins [13]. Strong associations have been found between Rheumatoid Arthritis and the segment of the MHC class III region which includes these LY-6 members. The characterisation of these transcripts is of great relevance for the understanding of human diseases.

LY-6 superfamily members are cysteine-rich, generally GPI-anchored, cell surface proteins which have definite or putative immune-related roles [14]. Among these LY-6 MHC class III region genes *LY6G5B *and *LY6G6D *showed a particular behaviour in the regulation of their expression [12], involving an intron retention event. The intron retained is the first in the open reading frame and interrupts the protein just after the signal peptide introducing a premature stop codon. The presence of a premature block to transcription in this position should cause this intron-retaining transcript to undergo Nonsense Mediated Decay (NMD) [15-17]. However, this transcript is present and is generally more abundant than the correctly spliced partner in all cell lines and tissues analysed [12]. Intron retention is the least characterised event of all alternative splicing types, mainly because of the exclusion of this phenomenon in many studies, due to the difficulty to differentiate it from genomic DNA or incompletely-processed transcripts. Moreover, it is not relevant to functional studies due to the introduction of premature stop codons. A number of studies indicate that up to 15% of human genes present at least one intron retention event, and that at least 22% of all informative intron-retention events are also present in the mouse transcriptome [18]. Finally, many intron retention events occur in the 5' and 3' Untranslated Regions (UTR) [18], that are still incompletely characterised for most genes.

Interestingly, we were also able to detect the presence of the exons of the *LY6G5B *and *LY6G6D *genes in transcripts derived from the upstream genes in the chromosome. This phenomenon, known as Transcription Induced Chimerism (TIC), or Tandem Chimerism is still largely unknown in its mechanism, but it is being promoted as a novel way to increase combinatorial complexity of the proteome [19-21]. Recent bioinformatics analyses, partially supported by experimental validation, show that this phenomenon could be quite frequent (up to 4–5% of the tandem gene pairs in the human genome) [20]. There are also cases of TIC described in which a chimeric protein can be detected or a logical function inferred [22-24].

Here we report a precise description and quantification, of the transcripts generated by intron retention events, of the MHC *LY6G5B *and *LY6G6D *genes. As these transcripts have a premature stop codon, they should be degraded quickly by Non-sense Mediated Decay. Nevertheless, they seem to be stable and even the most abundant transcript, especially in tissue samples. This could indicate that these mis-spliced forms are real transcripts which could have potential regulatory functions. In addition, we show that the *LY6G5B *and *LY6G6D *genes can form chimeric transcripts with adjacent genes.

## Results

### LY6G5B and LY6G6D transcript expression

**Figure 1:**
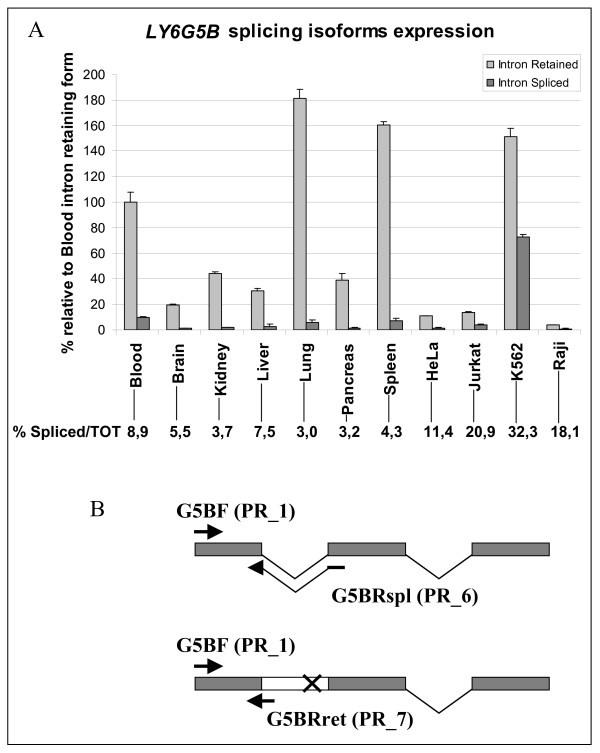
**Differential real time RT-PCR assay for the two LY6G5B splice isoforms in a panel of human tissues and cell lines.** (A). Data are expressed in relation to the percentage of the intron-retained form expressed in blood. Numbers below the graph represent the percent of the correctly spliced isoform of LY6G5B relative to the total expression of the gene in each sample. PCR reactions were run in triplicates. (B) Schematic representation of the primer design for the differential assay. Forward primer (PR_3) is shared while reverse primers (PR_6 and 7) share only 4 nucleotides at the end of the first exon. X indicates the premature stop codon.

The *LY6G6D *and *LY6G5B *genes express a small first intron (98 and 148 nucleotides, respectively) in the open reading frame which tends to be retained in the majority of cell lines and tissues, both in human and mouse RNAs [12]. To better understand the regulation of expression of these genes we performed a detailed analysis of the two transcripts of the *LY6G5B *gene and their relative levels in some cell lines and tissues by real time RT-PCR. The results shown in Figure 1 confirm that the intron-retaining form is the most abundant in all the samples analysed. The highest expression of this mis-spliced form was detected in lung, spleen, and in whole blood, and the K562 cell line. K562 cells also had the highest expression of the correctly spliced form.

### Transcript localisation, mRNA stability and NMD escape

**Figure 2:**
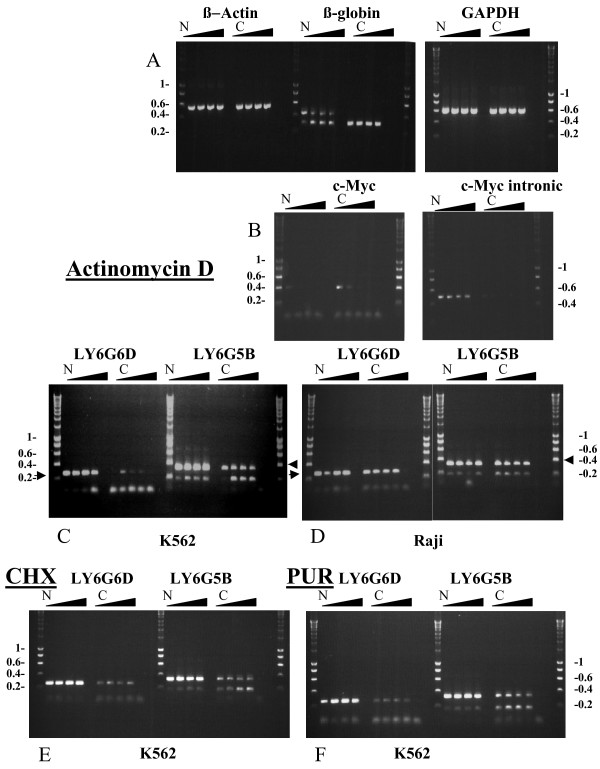
**RT-PCR results of Actinomycin D (A, B, C, D,) Cycloheximide (CHX) (E), and Puromycin (PUR) (F) treatment.** As controls the β-Actin, β-Globin and GAPDH genes (A) and the c-Myc transcript and c-Myc intron (B) were tested using nuclear (N) or cytoplasmic (C) RNA from the K562 cell line. Time points were as follows: Nuclear (N) Time 0, 1, 2, 4 hrs,, Cytoplasmic (C) Time 0, 1, 2, 4 hrs. For the LY6G5B and LY6G6D samples (C, D, E, F) the higher sized product is always the intron retaining one (indicated by an arrow) while the lower one is the correctly spliced form. Markers are indicated in Kb.

To ascertain that the intron retaining transcript we detected in our samples is not a splicing intermediate but a fully processed and exported mRNA, we performed a differential extraction of the nuclear and cytoplasmic RNA followed by RT-PCR (Figure 2). Correctly spliced mRNAs should be more stable after termination of transcription than the non-fully spliced forms containing a premature termination codon (PTC). To analyse whether that is the case for the mis-spliced forms, cells were treated with Actinomycin D, a transcriptional inhibitor, to measure mRNA decay rates and differences between the splice forms of the *LY6G6D *and *LY6G5B *genes and of other control genes.

Treatment with Actinomycin D indicated that these mis-spliced forms were stable in the cytoplasm, though there did appear to be cell-specific differences in stability for the *LY6G6D *gene, which seemed to be unstable in the K562 cell line, but stable in the Raji cell line (Figures 2C and 2D). Interestingly, the mis-spliced (334 bp) form of the *LY6G5B *gene was also more abundant in the cytoplasmic RNA fraction of Raji cells, relative to the correctly spliced form (187 bp), compared to K562 cells (Figures 2C and 2D). In addition, there appeared to be no differences in stability between the mis-spliced and correctly spliced forms of *LY6G5B *as there was no obvious decay of either form after transcription was stopped. For *LY6G6D *we were only able to amplify the mis-splced 269 bp form, and not the 178 bp properly spliced form (Figures 2C and 2D). As control RNAs we used the housekeeping genes β-actin and GAPDH for evaluating abundance and RNA quality and the β-globin RNA as a control of a stable RNA transcript (Figure 2A) observing that the RNA was not degraded by the treatment or by the RNA isolation procedure. We also used c-Myc as a control for unstable mRNA transcripts and the intron of c-Myc as a control for genomic contamination of the cytoplasmic RNA fraction (Figure 2B). The decay of the c-Myc transcript indicated that the Actinomycin D treatment was effective and the lack of c-Myc intronic product in the cytoplasmic RNA fraction showed there was no genomic contamination in the cytoplasmic fraction. The β-globin amplification showed genomic, or splicing intermediates, in the nuclear RNA fraction (upper band of 440 bp) in addition to the correctly spliced form (lower band of 320 bp), while in the cytoplasmic RNA fraction we could only detect the correctly spliced form (320 bp), indicating no genomic contamination in the cytoplasmic fraction. In the case of β-actin and GAPDH only the correctly spliced forms of 548 bp and 612 bp, respectively, were obtained in the amplifications of the nuclear and cytoplasmic RNA fractions. No genomic products (which would be 1123 bp and 2858 bp, respectively) were observed in either RNA fraction. All the controls were also performed with Raji cell extracts and the same results were obtained (data not shown). The results from all these experiments indicate that the unspliced LY6 transcripts are real transcripts and not due to genomic contamination.

**Figure 3:**
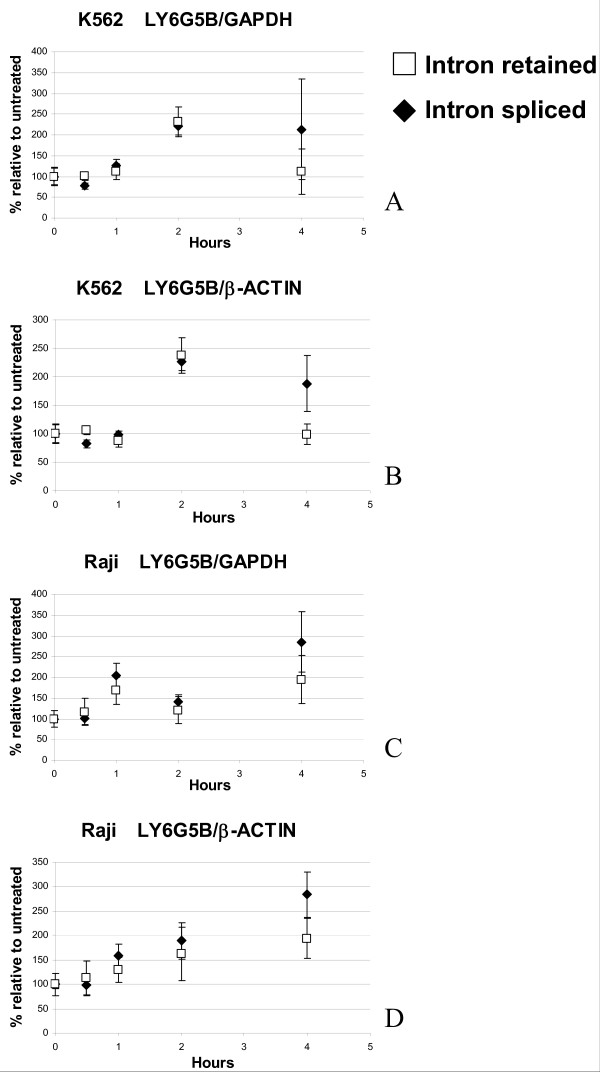
**Real Time-PCR to quantify the amount of intron-retained and intron-spliced forms of the LY6G5B transcripts in K562 (A, B) and Raji (C, D) cell lines harvested at times 0, 0.5, 1, 2 and 4 hrs post treatment with Actinomycin D.** Data are expressed as the percentage of each form at time 0 with no treatment. PCR reactions were run in triplicate.

We confirmed this experiment by measuring the levels of the two *LY6G5B *transcripts by a real time-PCR assay (Figure 3). In this case expression levels of the two splicing isoforms were normalised [25] to either GAPDH (Figure 3A and 3C) or β-Actin (Figure 3B and 3D) levels in K562 and Raji cells. As the transcripts for these two control genes also have their own kinetics of degradation we cannot measure an absolute stability of *LY6G5B*, but a relative stability compared to the control genes. In all cases we observed an increase in the relative expression of the *LY6G5B *isoforms with time, allowing us to conclude that the *LY6G5B *transcripts are more stable than Actin and GAPDH mRNA (Figure 3).

As the intron-retaining transcripts of the two genes have a PTC they should be subjected to degradation by the NMD machinery. To see whether this process acts on the non-coding transcripts we looked at the effect of translational blockage on stability, as translation of the mRNA has been shown to be required for NMD, probably for recognition of PTCs. Cycloheximide inhibits the peptidyl-transferase on the large subunit of the eukaryotic ribosome, while puromycin is a tRNA analogue that causes premature chain termination. If the NMD pathway was in some way acting on the mis-spliced transcript, we would expect to see an increase in the intron-retaining form relative to that of the correctly spliced form (observed as an increase in PCR product). K562 cells were treated with either cycloheximide or puromycin and showed no increase in stability of the mis-spliced forms of *LY6G5B *and *LY6G6D *relative to the correctly spliced forms, suggesting that these mis-spliced transcripts are not subject to NMD (Figures 2E and 2F).

### Luciferase assay

**Figure 4:**
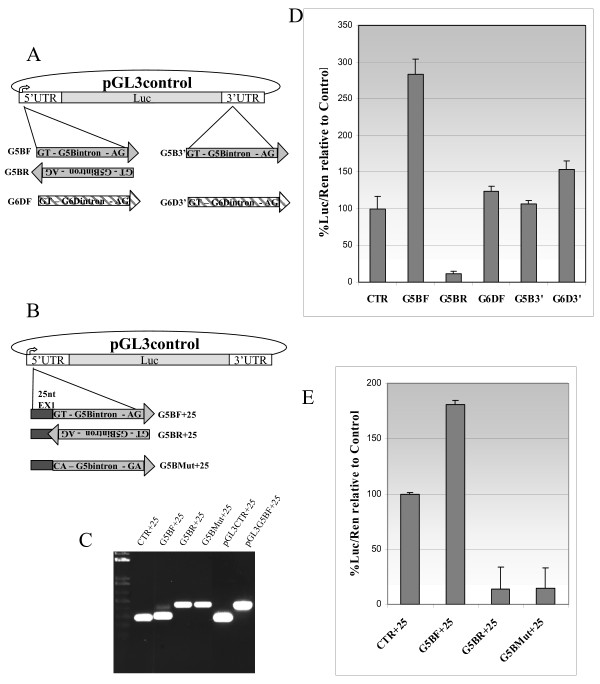
**Luciferase assay with cloned retained introns. Schematic representation of the constructs used in the luciferase assay (A and B).** Luc refers to the firefly luciferase ORF while GT- to the exon-intron boundary and -AG to the intron-exon boundary. 3' indicates the position of insertion relative to the luciferase ORF. In B dark grey rectangle represents the 25 nt of LY6G5B exon 1 added to the construct. (C) RT-PCR with primers PR_36, 37 and 38, and PR_41 on cDNA derived from transfected cells to identify spliced transcripts from the various plasmids. pGL3CTR+25 and pGL3G5BF+25 represent positive controls where the PCR reaction was performed on the original plasmids. Results of the luciferase assay (C and E) expressed as the relative response ratio, normalised for Renilla luciferase signal, and relative to the control.

To understand whether the intron was retained for its own features such as weakly recognised by the splicing machinery, or in a regulated fashion dependent on the molecular environment, the first introns of *LY6G5B *and *LY6G6D *were cloned in the 5' and 3' UTRs of a pGL3 control luciferase plasmid (Figure 4A). As some splicing factors inhibiting 5' splice site recognition, like hnRNP-F/H, have been described to bind just upstream of the 5' splice site [26,27], we also created some constructs containing 25 bases of the first exon just upstream of the intron, to generate part of the natural sequence context (Figure 4B). The results obtained showed, surprisingly, that the intron is fully spliced in this artificial mRNA assay, as the size of the amplified product from RNA derived from cells transfected with the G5BF+25 plasmid, which contains the intron in the 5'UTR of the luciferase construct, is the same as the control CTR+25 (Figure 4C). When the luciferase assay was performed in these transfected cells, surprisingly we found that the luciferase signal was increased more than 2.8 fold when the intron of *LY6G5B *(G5BF) was inserted in the 5' UTR in the correct orientation, probably due to a strong stabilisation effect [28] when splicing occurs in this position (Figure 4D). This effect is also observed, although to a lesser extent, in the construct with the 25 bp of the *LY6G5B *first exon (G5BF+25) (Figure 4E) and is not observed with the *LY6G6D *intron (G6DF) (Figure 4D). Constructs that retain the intron, the one with the intron in the reverse orientation (G5BR) (Figure 4A and 4D) and G5BR+25 (Figure 4B and 4E) and the other with the mutated intron boundaries (G5BMut+25 in Figure 4D), show a markedly lowered expression of the luciferase. This could be due to the presence of other start codons in the intron in both orientations, which are not in phase with the luciferase ORF.

### EST analysis of the LY6G5B and LY6G6D genes

To better define the expression pattern of these genes in order to characterise the UTRs and to support our data on intron retention in the expressed transcripts, we performed a detailed EST analysis on the two genes. We previously [12] presented an EST analysis, but at that time only two ESTs were found for each of the two human genes (Acc. no AI800033 and AA535815 for *LY6G6D*; AI446559 and R79468 for *LY6G5B*), making it difficult to completely define the gene. For *LY6G6D *there is only one additional EST from the 2002 analysis [12] that aligns with the whole intron-retaining transcript; while the previously described ESTs only cover exon 3 of the gene. Thus, there is still no EST corresponding to the correctly spliced and protein expressing form, despite the fact that we could find the correctly spliced form in many human tissues [12]. This is most likely due to the small number of ESTs, especially for low expression transcripts, and to the limitations of the EST data bank that often presents only partial sequences.

**Figure 5:**
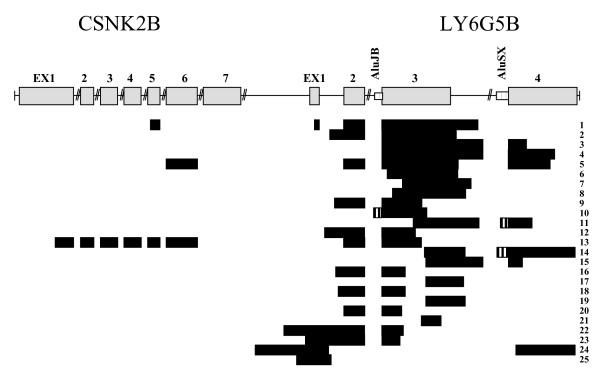
**Alignment of LY6G5B EST sequences (black boxes) on the genomic region comprising the genes CSNK2B and LY6G5B.** Grey boxes represent exons and its dimension is proportional to the real length. Striped boxes represent the position of the Alu repetitive elements. Numbers on the right are referred to in Table 1.

In contrast, our recent analysis has revealed that for *LY6G5B *there are a total of 25 ESTs covering the whole gene, part of the 5' UTR and 3'UTR (Figure 5 and Table 1). An additional intron and a fourth exon are probably present in the 3'UTR, as many ESTs map in that region. At least 6 out of the 23 ESTs containing exon 3 continue for about 200 bases after the stop codon where the alignment is interrupted, then most of them align with a region about 750 bases downstream of the end of the exon, just downstream of an AluSX element. Another Alu repeat of the family AluJB is contained in the second intron. Two ESTs (BQ447231 and BQ181819) contain a final polyA signal that defines a fourth and last exon of at least 320 bases. We could also define a large 5'UTR for the *LY6G5B *gene, as one EST (BQ181819, number 24 in Figure 5) extends up to 315 bases upstream of the translation start site. Among all ESTs we only found two (BF820976 and CT001189, corresponding to numbers 23 and 22 in Figure 5) which cover the entire exon 1, intron 1, exon 2 region. Surprisingly three ESTs (CF264683, BX363221 and BX363222, corresponding to numbers 1, 5 and 13 in Figure 5) of the human *LY6G5B *transcript align with some exons of the gene found upstream in the genomic sequence, the Casein kinase II beta subunit (*CSNK2B*) (Figure 5), suggesting the presence of transcriptional induced chimerism.

**Table 1:** LY6G5B ESTs.

ACC. NO	TISSUE	EX1	INT1	EX2	EX3	CHIM	NO. Fig 5
BX505974	ADULT				X		3
BX644716	ADULT				X		10
CF264683	BRAIN GLIOBLASTOMA	1/2		X	X	X	1
AW852310	COLON				X		6
BE697652	COLON				X		21
BG999849	HEAD			X	X		20
BE710662	HEAD-NECK		X	X	X		16
BX363221	HELA CELLS			X	X	X	5
BX363222	HELA CELLS			X	X	X	13
BF820976	KIDNEY TUMOR	X	X	X	X		23
AW581385	LEIOMIOS				X		17
AW608502	LEIOMIOS				X		19
CB132428	LIVER		1/2	X	X		2
BP305317	MACROPHAGE				X		11
BI041577	NERVOUS TUMOR				X		8
BM687278	OPTIC NERVE		X	X	X		12
BQ447231	OSTEOARTHRITIC CARTILAGE				X		14
BQ181819	OSTEOARTHRITIC CARTILAGE	X	X				24
R79468	PLACENTA				X		15
BI052431	PLACENTA			X	X		18
BG015680	PLACENTA NORMAL		1/3	X	X		9
BI049844	PLACENTA NORMAL	X	X				25
BE768359	PROSTATE TUMOR				X		7
BX437637	THYMUS				X		4
CT001189	T-LYMPHOCITES	X	X	X	X		22

Columns are marked with a X if the EST contains the whole sequence. CHIM column is marked if any sequence of CSNK2B is present. 1/2 and 1/3 mean that only this proportion of the sequence is present in the EST.

### Chimeric transcripts

**Figure 6:**
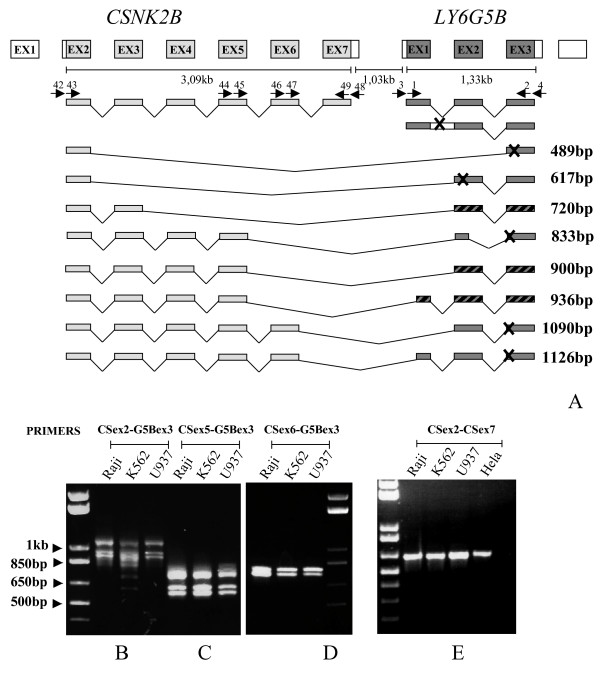
**Nested RT-PCR to characterise chimeric products between CSNK2B and LY6G5B. (A) Schematic representation of the cloned and sequenced products on the genomic structure.** Arrows represent primers used whose number corresponds with the number in Table 2. On the right are reported the length of the product as represented in the figure. Dashed boxes indicate inframe protein sequence. Each set of primers was tested in the indicated cell lines: primers from exon 2 (B), exon 5 (C) or exon 6 (D) of CSNK2B with exon 3 of LY6G5B. In E we report the RT-PCR for the CSNK2B ORF, from exon 2 to 7, is shown. X indicates premature stop codon.

To prove the presence of the chimeric transcript for *LY6G5B *we performed RT-PCR using primers from the second, fifth and sixth exons of the *CSNK2B *gene and the third exon of *LY6G5B *(Figure 6A). We found a defined pattern of bands (Figure 6B) in Raji, K562 and U937 cells whose sequences represent many combinations of exons from the two genes (Figure 6A). Three main bands of 1090, 936 and 900 bp were found when the nested RT-PCR was performed for the whole chimeric transcript. The first (1090 bp) corresponds to exons 2 to 6 of *CSNK2B *spliced to exons 2 and 3 of *LY6G5B *though the resulting chimeric transcript is not in frame with the *LY6G5B *ORF. The other two bands of 936 bp and 900 bp correspond to exons 2 to 5 of *CSNK2B *spliced to the last 36 nucleotides of exon 1 and exons 2 and 3 of *LY6G5B *(936 bp), or directly to exons 2 and 3 of *LY6G5B *(900 bp) which maintain the *LY6G5B *ORF. Other less abundant transcripts were also detected (see Figure 6A) which were confirmed when primers from exons 5 or 6 of *CSNK2B *were used in the PCR reactions (Figures 6C and 6D). Amplification under the same conditions of the *CSNK2B *gene using primers from exons 2 and 7 resulted in the appearance of a single band of 645 bp (Figure 6E) corresponding to only one RNA form, the one described in the literature [29].

**Table 2:** Primers

**Primer**	**Name**	**Sequence**
**PR_1**	G5Bh-int-F	ATGAAGGTCCATATGCTTGTAGG
**PR_2**	G5Bh-int-R	CATAATAGATGGTGATCACCATGCAC
**PR_3**	G5Bh-UTR-F	CTCAGGAACTGCCCATCTCCCCAG
**PR_4**	G5Bh-UTR-R	GGGTGTTACAGAAAGATATTTGCAC
**PR_5**	G5Bh-ex3-R	TCAGGAAGGGTGAGGTGTCAAG
**PR_6**	G5B real time_spl	TGTCGGGAACAGGAACCTTT
**PR_7**	G5B real time_ret	GGGCCCCACTTACCCTTT
**PR_8**	G6Dh-int-F	ATGAAACCCCAGTTTGTTGGG
**PR_9**	G6Dh-int-R	GGTTTCCAGGTAGCTTGATCTGTTCC
**PR_10**	G6Dh-UTR-F	GGTCCTGACACGGGCAGACTGC
**PR_11**	G6Dh-UTR-R	GTTCCCTTCTCTACTCCTACTCCCC
**PR_12**	G6Dh-ex3-R	CTATCCGCTCCACAGTCCTGG
**PR_13**	b-actin-F	CTTCGCGGGCGACGATGC
**PR_14**	b-actin-R	TGGTGGTGAAGCTGTAGCC
**PR_15**	b-globin-F	ATGGTGCATCTGACTCCTGAGG
**PR_16**	b-globin-R	CTGAAGTTCTCAGGATCCACGTG
**PR_17**	GAPDH-F	ATGGGGAAGGTGAAGGTCGGAGTC
**PR_18**	GAPDH-R	GCGGCCATCACGCCACAGTTTC
**PR_19**	cMYC RT-F	ATGCCCCTCAACGTTAGCTTCACC
**PR_20**	cMYC RT-R	CAGGACTTGGGCGAGCTGCTG
**PR_21**	cMYC intr-F	CAGGCTTAGATGTGGCTCTTTGGG
**PR_22**	cMYC intr-R	TTCGCCTCTTGACATTCTCCTC
**PR_23**	GAPDH_REALT-F	ATCAGCAATGCCTCCTGCAC
**PR_24**	GAPDH_REALT-R	GGCATGGACTGTGGTCATGA
**PR_25**	ACTIN_REALT-F	GATCATTGCTCCTCCTGAGC
**PR_26**	ACTIN_REALT-R	CCTGCTTGCTGATCCACATC
**PR_27**	g5biF hindIII	CGGAAGCTTGTAAGTGGGGCCCAGGGGCAGGGAG
**PR_28**	g5biR hindIII	CGGAAGCTTCTGGGGAGGCAGAAGGAGGGATGGA
**PR_30**	g6diF hindIII	CGGAAGCTTGTAAGGAGGCGGCCAGCTAGCTTCT
**PR_31**	g6diR NcoI	CGGCCATGGCTGGCAGGAGAAAAGAGGCGCTGGA
**PR_32**	g5biF PstI	CGGCTGCAGGTAAGTGGGGCCCAGGGGCAGGGAG
**PR_33**	g5biR EcoRV	CGGGATATCCTGGGGAGGCAGAAGGAGGGATGGA
**PR_34**	g6diF PstI	CGGCTGCAGGTAAGGAGGCGGCCAGCTAGCTTCT
**PR_35**	g6diR EcoRV	CGGGATATCCTGGCAGGAGAAAAGAGGCGCTGGA
**PR_36**	G5BF+25	GAAGCTTGGTGGGCTTCACAGTAGGAAAGGAAGCTTC
**PR_37**	G5BR+25	GAAGCTTCCTTTCCTACTGTGAAGCCCACCAAGCTTC
**PR_38**	G5BMutF+25	CGGAAGCTTCTGCAGCAAAGTGGGGCCC
**PR_39**	G5BMutR+25	GCCAAGCTTGATATCTCGGGGAGGCAGA
**PR_40**	pGL3c39f	CTGCGATCTGCATCTCAATTAG
**PR_41**	pGL3c489r	ATATCGTTTCATAGCTTCTGCCA
**PR_42**	CSNKh-ext-ex1-2	CCGTCCAGCCGCTGACGTGAAG
**PR_43**	CSNK-int-ex2	ATGAGCAGCTCAGAGGAGGTGTC
**PR_44**	CSNK-ext-ex5	GACTTTGGTTACTGTCCTCGTGT
**PR_45**	CSNK-int-ex5	TGTGAGAACCAGCCAATGCTTCC
**PR_46**	CSNK-ext-ex6	GACATCCCAGGTGAAGCCATGG
**PR_47**	CSNK-int-ex6	CTCTACTGCCCCAAGTGCATGG
**PR_48**	CSNKh-ext-R	CAAAGACTGCAGGACAGGTGG
**PR_49**	CSNK-int-R	TCAGCGAATCGTCTTGACTGG
**PR_50**	G6Fh-ext-ex1	CAAGAGAACTTGGCAGGCTC
**PR_51**	G6Fh-int-ex1	CCCCATGGCAGTCTTATTCC
**PR_52**	G6Fh-ext-ex4	CCCTCTGTGCCCCTTCCACG
**PR_53**	G6Fh-int-ex4	GGGACATGCCTTGGATTCTG

**Figure 7:**
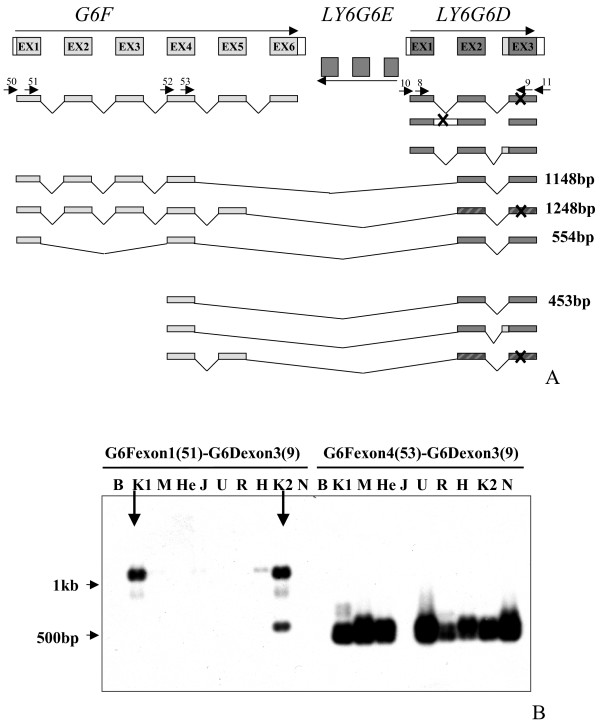
**Nested RT-PCR to characterise chimeric products between G6F and LY6G6D.** (A) Schematic representation of the cloned and sequenced products on the genomic structure. Arrows represent the primers used, whose number corresponds with the number in Table 2. X indicates premature stop codon. Dashed boxes indicate inframe protein sequence. (B) Southern Blot of the PCR products to identify specific bands. Primers used in PCR on indicated cell lines: primers from exon 1 and exon 4 of G6F with exon 3 of LY6G6D. (B:143B, K1:K562, M:Molt4, He:HeLa, J:Jurkat, U:U937, R:Raji, H:HL60, K2:K562 of different origin than K1, N:negative)

Interestingly, when we performed BLAST analysis of the LY6G6D protein we found a perfect match to part of the human megakaryocyte-enhanced gene transcript 1 (MEGT-1) protein (E value of 2e^-50^; data not shown) already deposited in GeneBank as a fusion gene with the accession number AF195764. The MEGT-1 protein also showed a perfect match to the translation of exons 1–4 of the *G6F *gene. The *G6F *gene (AF129756), which is expressed in platelets [30,31], encodes a novel member of the immunoglobin superfamily [32]. *G6F *consists of six exons, *LY6G6D *of three exons and they are separated by the three exons of *LY6G6E*, which are theoretically transcribed in the reverse orientation to *G6F *and *LY6G6D *(Figure 7A). We then confirmed that the MEGT-1 transcript consists of exons 1–4 of *G6F*, which then splice to exons 2 and 3 of *LY6G6D*, skipping exons 5 and 6 of *G6F*, all of *LY6G6E *and exon 1 of *LY6G6D *(Figure 7A).

To examine the expression patterns of the *G6F/LY6G6D *chimera, two different RT-PCR reactions were performed in a number of different human cell lines, one amplifying from exon 1 of *G6F *to exon 3 of *LY6G6D*, and the other amplifying from exon 4 of *G6F *to exon 3 of *LY6G6D*, to see whether a shorter transcript was also present (Figure 7A) as exon 4 of *G6F*, which normally codes for the transmembrane region of the protein, could act as a signal peptide. Southern blot analysis (Figure 7B) showed that there are different transcripts produced from the *G6F *exon 1 – *LY6G6D *exon 3 amplification (long chimera). A ~1.2 kb doublet is present mainly in the K562 cell line as well as a faint band at ~900 bp and a band at ~500 bp (Figure 7B). The 1.2 kb band is also present faintly in the HL60, Jurkat and Molt4 cell lines corresponding to the expected transcript, consisting of exons 1–4 of *G6F*, spliced to exons 2 and 3 of *LY6G6D *(1148 bp). This would translate to give a chimeric transcript containing the Ig domain and the transmembrane region of G6F fused to the LY-6 domain of LY6G6D. The upper band of the ~1.2 kb doublet consists of exons 1–5 of *G6F *spliced to exons 2 and 3 of *LY6G6D *(1248 bp) which translate to a G6F protein including the transmembrane domain and a cytoplasmic domain due to an inframe amino acid sequence encoded by exons 2 and 3 of *LY6G6D*. Other secondary bands have been cloned and are represented in figure 7A, but none retain the LY-6 ORF.

Splice variants of the expected ~450 bp shorter form of the chimera (exon 4 of *G6F *and exons 2–3 of *LY6G6D*) were seen in all cell lines except HeLa, with the strongest signal in the K562, Jurkat, Molt4, and 143B cell lines (Figure 7B). After sequence analysis three different splice variants were found; including the expected form of 453 bp which results in a signal peptide followed by the LY-6 domain. Other secondary bands are shown in the figure but they lose the LY-6 ORF and do not match with any known or predicted protein.

## Discussion and conclusion

During the last fifteen years a major effort has been centred on describing the number and the position of all human genes and this aim has been achieved through a combination of advances in sequencing technologies and bioinformatics prediction programs. Nevertheless, there are still a considerable number of genes that remain uncharacterised, and even more where we know virtually nothing of their transcriptional control including their differential tissue expression and splicing regulation. In addition, bioinformatics tools are becoming very accurate in organising biological information and predicting the structure and function of genes, but sometimes adjustments in data processing can affect the conclusions which can be drawn. For instance, in the case presented here, the fact that non-coding transcripts sometimes are excluded from analyses and databases, to avoid the risk of considering non-fully processed intermediate transcripts, could cause a significant underestimation of the frequency of intron retention transcripts.

Alternative splicing is understood as a regulatory process, contributing to biological complexity through its ability to control the expression of proteins. An mRNA variant has been defined as being 'functional' if it is required during the life-cycle of the organism and activated in a regulated manner. In some cases, functional splice forms may not even be required in their own right, but their production is required to regulate active protein levels. Moreover, the meaning of 'required' can be generalized by defining functional splicing as that which conveys a selective advantage [33].

The extent to which splicing has a role in disease, as either a direct cause, a modifier or a susceptibility factor, continues to be defined. Advances in several areas will clarify the roles of splicing in disease and reveal the mechanisms involved, and will allow routine application of the knowledge gained toward diagnosis and treatment. One major advance will be to develop the ability to predict splicing outcomes associated with genetic variants and disease-causing mutations [28]. Important insights will be gained from the full characterization of the human transcriptome, which would provide a catalogue of all the splice variants expressed from each gene and identification of the isoforms that predominate in specific cell types and tissues. This is a significant challenge that will have a huge benefit, not least being the ability to design microarrays that can be applied for the quantitative assessment of all splice variants. Finally, another key direction for the future will be the application of genome-wide microarray assays to assess splicing differences associated with normal variation and with disease. Alternative-splicing signatures are likely to provide a useful diagnostic and prognostic tool for many diseases. As for other challenges, the tools that are required to meet this challenge are developing rapidly [1].

Here we have presented a detailed transcriptional analysis of the *LY6G5B *and *LY6G6D *genes. The striking feature which was observed in a first analysis was the intron retention event that generated a non-coding transcript. In fact, the protein coding transcript of these genes is the one described in databases, but we found that, by the retention of the first intron, its expression is nearly completely silenced. This event seems not just to be an inherent feature of the introns because they are spliced correctly when the introns are inserted in an artificial construct, such as the luciferase expression plasmid used in this study, although they are also able to markedly stabilise the artificial transcript. In order to be retained the introns must be in the genomic environment of their particular Ly-6 genes, and for this reason might have a regulatory role in these genes. In addition, by real time RT-PCR we found that the retained and spliced forms are differentially expressed in tissues, indicating an active regulation of the non-coding transcript. We propose that when the gene is expressed the intron retention event could be regulated by a cis-acting element, acting on the processing machinery. It could be possible that this block in the expression is released only in a precise physiological response or at a particular developmental stage or in a specific pathologic process. Related to this, Yan et al. (2005) [34] showed a novel gene *Saf*, transcribed from the opposite strand of a noncoding intronic region of the *Fas *gene, that acts in cis and regulates alternative splicing forms of Fas. In addition, noncoding RNAs are abundantly transcribed from the introns of 74% of all RefSeq genes [35] and could be involved in regulation of alternative splicing in response to physiological and pathologic conditions.

Another possibility is that the chimera represents an alternative way for the expression of the protein, that borrows the promoter of the preceding gene to be expressed and then by a combination of transcript and protein processing reaches its final expressed form. In the case of the *G6F-LY6G6D *chimera, for example, the MEGT-1 protein would encode two extracellular Ig domains, a transmembrane segment and then the LY6G6D LY-6 domain intra-cellularly. Interestingly, the fact that exon 4 of *G6F *could encode a signal peptide starting with a methionine residue and that there is a long intron between exons 3 and 4 in *G6F*, suggests the presence of a promoter region specific for the shorter transcript. The possibility of an alternative promoter in this case is also supported by the expression results, as the short chimera seems to be expressed in nearly all analysed cell lines while the long chimera is expressed mainly in the K562 cell line, which is also the only cell line that expresses *G6F*.

The particular behaviour of these genes could be more general, strengthening the importance of a detailed transcriptional analysis of every gene, because their physiological and pathological roles could be based on unexpected forms of expression regulation. Susceptibility to human diseases is associated with genes in the MHC class III region. Microsatellite and SNP genotyping studies have attempted to fine map the location of these genes, finding strong associations between Rheumatoid Arthritis and a 126 Kb region in the MHC class III region, which include these LY-6 members. The characterization of the LY-6 transcripts is of great relevance for the understanding of human diseases.

## Methods

### Cell cultures and treatments

The cell lines Jurkat (T cell), U937 (monocyte), Raji (B cell), HL60 (monocyte), Molt4 (T cell), and K562 (undifferentiated erythroleukaemia) were grown in RPMI medium while Hek293T (embryonic kidney), HeLa (epithelial), 143B (TK-) (osteoblast) were grown in DMEM, all with 10% (v/v) foetal bovine serum (FBS), 100 IU/ml penicillin and 100 μg/ml streptomycin. Cells to be treated with Actinomycin D (5 μg/ml), Puromycin or Cycloheximide (both 300 μg/ml) were plated at 5 × 10^6 ^cells and grown for 24 hours prior to the addition of the reagent. Cells were incubated with the reagent for 0, 30 min, 60 min, 120 min and 240 min prior to harvesting.

### RNA extraction, RT-PCR and Real Time-PCR

The SV RNA isolation kit (Promega) was used for RNA isolation from frozen pellets containing 5 × 10^6 ^cells followed by DNAse treatment. Human tissue RNAs were obtained from BioChain^® ^(USA)  through one of their Europe distributor "ams"  (UK). One μg of total RNA obtained from each sample was used for oligo-dT primed cDNA synthesis which was performed using the ImProm Reverse Transcription System (Promega) in a 20 μl reaction volume following the manufacturer's instructions. Initial experiments were performed to check for the presence of these gene transcripts in the cytoplasmic fraction of the cell and to optimise the fractionation procedure (data not shown). The fractionantion was performed using the Qiagen RNeasy mini kit cytoplasmic RNA extraction protocol. We improved the method for our cell types, by using 5 × 10^6 ^K562 cells and 1 × 10^7 ^Raji cells. We spun intact cells at 2000 rpm and lysed them in modified RLN buffer (10 mM Tris pH8, 100 mM NaCl, 1.5 mM MgCl_2_, 0.5% NP40, 1000 U/ml RNasin and 1 mM DTT). The cytoplasmic-nuclear fraction was separated by spinning at 1800 rpm for 2 minutes. The cytosolic fraction was taken and RLT buffer added and processed as described in the Qiagen protocol. To the nuclear fraction was also added FLT buffer and processed as described in the protocol. For Reverse Transcription-PCR (RT-PCR) 1 μl of cDNA was used in each PCR reaction. All RT-PCR reactions contained 2 mM MgCl_2_, 0.8 mM dNTPs, 0.4 μM each primer and 0.75 U Taq polymerase (Roche) in a 25 μl reaction volume. The PCR conditions were as follows: 95°C for 2 min followed by 35 cycles of 95°C for 45 s, 60°C for 30 s, 72°C for 30 s, followed by 72°C for 5 min. The primers used for b-actin were PR_13 and PR_14, for b-globin PR_15 and PR_16, for GAPDH PR_17 and PR_18, for cMyc PR_19 and PR_20, and for cMyc-inronic PR_21 and PR_22. The primers used for the amplifications of LY6G6D were PR_8 ad PR_12 and for LY6G5B were PR_1 and PR_5.

Real-time RT-PCR for *LY6G5B *was performed by using SYBR^® ^green PCR master mix and the ABI PRISM^® ^7700 sequence detection system (Applied Biosystems). Primers for real-time RT-PCR were designed for the differential quantification of the intron retention event, with a common forward primer and two reverse primers, one spanning the exon-intron junction for the intron retaining form and the other spanning the exon-exon junction for the correctly spliced form (PR_1, 6 and 7, respectively). As the intron retaining transcript cDNA does not differ at all from the genomic DNA and the correctly spliced form differs for only 148 bases of the intron, we always performed a RT minus (RT-) reaction for each sample (a reverse transcription reaction identical to the one described in the previous section, but without adding the reverse transcriptase) to check for lack of amplification, or consistently later amplification than the corresponding RT-treated sample (more than ten cycles of difference). Quantifications were always normalised using endogenous control GAPDH (PR_23 and 24) or β-actin (PR_25 and 26). To compare levels of the two isoforms in the same sample we had to perform an absolute quantification of the two isoforms in each sample [25]. To achieve this, we generated standard dilution curves. The two splicing isoforms of the *LY6G5B *gene were first amplified by nested PCR from K562 cDNA with the primers PR_1, 2, 3 and 4, then purified from a gel and cloned into the pGEM-T plasmid (Promega). The plasmid DNA was isolated, sequenced, then quantified precisely and diluted to the same copy number per millilitre. Serial dilutions of 1 in 5 volumes were then run in a real time RT-PCR assay with the primers PR_1, 6 and 7 generating the final curve to interpolate results from the cDNA samples.

### Luciferase assay

The control plasmid pGL3 (Promega) was modified to contain an insertion site (*Pst*I and *Eco*RV) in the 3'UTR of the firefly luciferase open reading frame (ORF). This was used as the starting plasmid for all the constructs in Figure 4A. Constructs containing introns were made by PCR amplification of the intron from genomic DNA using gene specific primers containing different restriction sites: *Hin*dIII for the *LY6G5B *intron (PR_27, 28), *Hin*dIII and *Nco*I for the *LY6G6D *intron (PR_30, 31) for the 5'UTR cloning, and *Pst*I and *Eco*RV for the 3'UTR cloning (PR_32, 33, 34, 35). Either pGL4.11 or pHRL (Promega) containing the renilla luciferase ORF were used as control for transfection efficiency. In order to be able to determine the role of the 5' flanking region in the intron of *LY6G5B*, and to be able to look at the splicing of the intron by RT-PCR, we created another construct by inserting the synthetic oligonucleotide (PR_36, 37), representing the last 25 bases of the *LY6G5B *exon 1, in the *Hin*dIII restriction site of the pGL3 control plasmid described above. The same insert of the *LY6G5B *intron was inserted in this new control plasmid in both orientations as well as a mutated version of the intron where the GT...AG boundaries were mutated to CA...GA with the primers PR_38 and PR_39 and *Hin*dIII digestion. This plasmid was used as control for intron retention. All constructs were sequenced with primers PR_40 and PR_41 to confirm the presence of the correct sequence. For the transfection Hek293T cells (1 × 10^6^) were seeded in 60 mm dishes and 24 hours later were transfected with 2 μg DNA (Luciferase plasmid and Renilla plasmid in a 20:1 quantitative proportion), using PolyFect (Qiagen) following the manufacturer's instructions. Two days after the transfection, cells were harvested in PBS, then a tenth of the total cells for each plate were transferred to a 96-well plate in duplicate and luciferase and renilla activity were sequentially measured using the Dual-Glo™ Luciferase Assay System (Promega) kit. The remaining cells were pelleted and the RNA extracted to be quantified by Real Time-PCR as described above.

### EST Data-base analysis

The EST analysis was performed for the two genes by submitting the sequence of each exon and of the first intron to a BLAST analysis in the human EST database at the National Center for Biotechnology Information (NCBI) , and the matching EST clones identified and aligned.

### Nested RT-PCR and Southern blot analysis for chimeric transcripts

For the *CSNK2B*-*LY6G5B *chimera the first round of PCR was performed with external primers in exons 1, 5 or 6 (PR_42, 44, 46) of *CSNK2B *and in the 3'UTR of *LY6G5B *(PR_4), and the second round using primers just downstream of the first round ones (PR_43, 45, 47 and 2). PCR reagents and conditions were the same as described above. For the second round 1 μl of a 1:10 dilution of the first round product was used as template. The PCR products were gel purified, cloned and sequenced (at least three clones for each band) then aligned to the genomic sequence. For the *G6F-LY6G6D *chimera the first round of PCR was performed with external primers in exons 1 or 4 of G6F (PR_50, 52) and in the 3'UTR of *LY6G6D *(PR_11), and the second round using primers just downstream of the first round ones (PR_51, 53 and 9). To verify that the products obtained were specific Southern blot analysis was performed. Specific products were detected using a probe consisting of exon 3 and the 3' UTR of *LY6G6D*, created by digesting IMAGE clone 2321242 (Accession number AI800033). The probe was labeled with fluorescein-11-dUTP as detailed in the manufacturer's protocol for ECL random prime labelling and detection systems, version II (Amersham Life Science). The signal was detected by Enhanced Chemiluminescence (ECL) and visualised by autoradiography. The PCR products of the K562 cell line for the PR_51 and 9 amplification (long chimera) and of the HL60 cell line for the PR_53 and 9 amplification (short chimera) were also cleaned (Qiagen PCR columns) and subcloned into the pGEM-T vector for sequencing.

### Statistics

The results are expressed as mean ± s.e.m. of the number of experiments indicated in the figure legends. The data were analysed by ANOVA and a probability level of P < 0.05 was considered to be statistically significant.

## Authors' contributions

VC carried out the majority of the research work, designed experiments, found and described the Ly6G5B-CSNK2B chimera, and helped on the first draft of the manuscript. MM performed the initial characterisation of the project, carried out the Actinomycin D and Cycloheximide experiments and described the Ly6G6D-G6F chimera. RDC participated in the design of the study and on the manuscript drafts. BA conceived the study, participated in the design and coordination of experiments and wrote the manuscript. All authors read and approved the final manuscript.
